# Genomic and functional divergence of *Staphylococcus aureus* strains from atopic dermatitis patients and healthy individuals: insights from global and local scales

**DOI:** 10.1128/spectrum.00571-24

**Published:** 2024-08-20

**Authors:** Zhongjie Wang, Claudia Hülpüsch, Bärbel Foesel, Claudia Traidl-Hoffmann, Matthias Reiger, Michael Schloter

**Affiliations:** 1 Research Unit for Comparative Microbiome Analysis, Helmholtz Munich, German Research Center for Environmental Health, Neuherberg, Germany; 2 Institute of Environmental Medicine, Helmholtz Munich, German Research Center for Environmental Health, Neuherberg, Germany; 3 Environmental Medicine, Faculty of Medicine, University of Augsburg, Augsburg, Germany; 4 CK CARE, Christine Kühne Center for Allergy Research and Education, Davos, Switzerland; 5 Chair of Environmental Microbiology, TUM School of Life Sciences Weihenstephan, Technical University of Munich, Freising, Germany; Institut Necker Enfants Malades, Paris, France

**Keywords:** *Staphylococcus aureus*, atopic dermatitis, gene content diversity, functional diversification, horizontal gene transfer, antibiotic resistance

## Abstract

Atopic dermatitis (AD) is the most common chronic inflammatory skin disease worldwide and is characterized by a complex interplay with skin microbiota, with *Staphylococcus aureus* often abnormally more abundant in AD patients than in healthy individuals (HE). *S. aureus* harbors diverse strains with varied genetic compositions and functionalities, which exhibit differential connections with the severity of AD. However, the differences in *S. aureus* strains between AD and HE remain unclear, with most variations seen at a specific geographic level, implying spontaneous adaptations rather than systematic distinctions. This study presents genomic and functional differences between these *S. aureus* strains from AD and HE on both global and local levels. We observed reduced gene content diversity but increased functional variation in the global AD-associated strains. Two additional AD-dominant clusters emerged, with Cluster 1 enriched in transposases and Cluster 2 showcasing genes linked to adaptability and antibiotic resistance. Particularly, robust evidence illustrates that the lantibiotic operon of *S. aureus*, involved in the biosynthesis of lantibiotics, was acquired *via* horizontal gene transfer from environmental bacteria. Comparisons of the gene abundance profiles in functional categories also indicate limited zoonotic potential between human and animal isolates. Local analysis mirrored global gene diversity but showed distinct functional variations between AD and HE strains. Overall, this research provides foundational insights into the genomic evolution, adaptability, and antibiotic resistance of *S. aureus*, with significant implications for clinical microbiology.

**IMPORTANCE:**

Our study uncovers significant genomic variations in *Staphylococcus aureus* strains associated with atopic dermatitis. We observed adaptive evolution tailored to the disease microenvironment, characterized by a smaller pan-genome than strains from healthy skin both on the global and local levels. Key functional categories driving strain diversification include “replication and repair” and “transporters,” with transposases being pivotal. Interestingly, the local strains predominantly featured metal-related genes, whereas global ones emphasized antimicrobial resistances, signifying scale-dependent diversification nuances. We also pinpointed horizontal gene transfer events, indicating interactions between human-associated and environmental bacteria. These insights expand our comprehension of *S. aureus*’s genetic adaptation in atopic dermatitis, yielding valuable implications for clinical approaches.

## INTRODUCTION

*Staphylococcus aureus* is a highly adaptable and versatile bacterium that can colonize and persist in diverse habitats in both human and animal hosts. The organism has a long evolutionary history as a multi-host commensal and opportunistic pathogen (1). *S. aureus* colonizes approximately 20%–30% of humans, persistently in the nose (2) and frequently in other body niches such as the skin, throat, axillae, and intestine (3, 4). *S. aureus* has also been isolated from the skin and mucous membranes of livestock (cattle, sheep, goats, etc.) and domestic animals like dogs and cats (5, 6), which might increase transmission frequencies between humans and other animals. *S. aureus* is recognized as a leading cause of a wide variety of clinical manifestations, from mild skin infections to serious invasive conditions, including impetigo, bacteremia, osteomyelitis, septic arthritis, pneumonia, and endocarditis (4, 7). Nevertheless, *S. aureus* strains also serve as non-pathogenic skin commensal, with certain strains even suggested to harbor beneficial immunomodulatory properties (8, 9).

The relationship between *S. aureus* and atopic dermatitis (as atopic eczema, AD), a chronic inflammation characterized by dry, itchy, and inflamed skin (10, 11), is particularly complex and multifaceted (12–14). AD is closely associated with other atopic comorbidities, such as food allergy, asthma, and allergic rhinitis (10, 15, 16). AD affects over 20% of children and 10% of adults in developed countries and shows an increasing global prevalence (17–20). Various new therapies for AD have been introduced, such as biologics like dupilumab and tralokinumab, and Janus kinase inhibitors including abrocitinib, baricitinib, and upadacitinib, along with conventional therapies like systemic glucocorticosteroids and ciclosporin (21). The skin of AD patients is characterized by a higher relative and absolute abundance of *S. aureus* compared to healthy individuals (HE), leading to a dysbiosis of the skin microbiome and significantly reduced overall microbial diversity (12, 22–24). This microbial imbalance plays a significant role in the pathogenesis and severity of AD. Dysbiosis weakens the skin barrier function, heightening susceptibility to external factors like irritants, allergens, and pathogens, leading to inflammation and infection (25). In addition, it disrupts the skin’s immune response, exacerbating inflammatory conditions and facilitating the colonization of harmful microbes (26). Consequently, microbial dysbiosis contributes to the chronicity and intensity of diseases like AD, highlighting the importance of reducing over-colonization by *S. aureus* and restoring microbial balance as a potential therapeutic strategy.

The presence of *S. aureus* on the skin of AD patients is positively associated with the severity of symptoms (23, 27, 28). The broken skin barrier in eczematous skin provides an entry point for the bacteria, leading to colonization and potential infection. When *S. aureus* invades the compromised skin barrier, it can lead to localized infections, further exacerbating the inflammation and itching associated with eczema (13, 29, 30). *S. aureus* strains can also develop resistance to antibiotics, making treatment more challenging or impossible (31, 32). For example, the widespread antibiotic resistance observed in methicillin-resistant *S. aureus* (MRSA) strains is largely attributed to horizontal gene transfer (HGT), with transduction, the process of transferring DNA between bacteria *via* bacteriophages, being the main mechanism (33, 34). Studies have demonstrated that MRSA strains evolve through HGT, gene duplications, and losses, particularly in virulence and antibiotic resistance genes (35, 36).

Previous evidence revealed that *S. aureus* strains isolated from AD and HE differ (32, 37–39), which indicates AD-associated strains can better adapt to inflamed skin, thus leading to a selective advantage in the AD context. A metagenomic study from the US demonstrated that *S. aureus* strains isolated from severe AD-affected patients caused greater inflammatory and immune responses *in vitro* than strains isolated from mild AD or HE individuals (37). A study from the UK reported varied prevalence of *S. aureus* clonal complexes between AD and HE groups, with over 80% of AD patient strains carrying a plasmid with the ß-lactamase gene for penicillin resistance, a trait absent in the HE group (38). A cohort study from Japan detected dysfunctional mutations of the accessory gene regulatory (*Agr*) quorum-sensing system predominant in the HE group, highlighting a connection between a functional *Agr* system and AD (40). Furthermore, recent insights showed that adaptive capsule loss *via capD* mutations*,* a gene crucial for initiating *S. aureus* capsular polysaccharide synthesis*,* was more common in AD globally (39), which provided a competitive advantage of improved adherence to AD skin. While this study showed the significance of investigating *S. aureus* strains at the global scale (39), most identified variances, however, have been on a regional level, suggesting their adaptations are rather spontaneous, instead of a general difference between AD and HE strains. The global perspective on whether genome differences exist between AD and HE or if their roles shift from commensal to pathogenic based on skin conditions remains unclear.

As AD skin represents a unique microenvironment, we hypothesize it might promote adaptive selection toward a homogeneous set of *S. aureus* strains. To investigate the genetic differences and underlying mechanisms driving the differentiation between *S. aureus* strains from AD and HE individuals, we analyzed 300 publicly available strains from nine countries. This global data set enabled a comprehensive *in silico* investigation of genomic and functional disparities, examining both micro-level (AD vs HE) and macro-level (global vs local) variations. Subsequently, we validated our findings using a local collection of 48 strains we isolated from AD and HE in Augsburg, Germany (41). Our analyses identified reduced gene content diversity of AD strains and geographically scale-dependent diversification mechanisms, thus providing a broader understanding of the gene content and functional variances of *S. aureus* from AD and HE at both global and local scales.

## RESULTS

### Data characterization

In our study, we analyzed a global data set of 150 AD strains from 4 countries and 150 HE strains from 9 countries, complemented by a local German data set of 33 AD and 15 HE strains. As shown in Table 1, the genome sizes averaged 2.80 Mb for both AD (range: 2.66–2.97 Mb) and HE (range: 2.69–3.12 Mb) strains. The GC content was approximately 32.82% for AD and 32.73% for HE. Genome assemblies displayed high average completeness (>99%) and minimal contamination (<0.3%). However, the two groups exhibited a distinct difference in the predicted average number of genes, with 2,588 genes in AD and 2,612 in HE strains. Additional details are given in Table S1.

**TABLE 1:** Statistics of all human-derived *S. aureus* assemblies

	AD			HE		
	Min	Mean	Max	Min	Mean	Max
Number of strains	150^*a*^ + 33^*b*^			150^*a*^ + 15^*b*^		
Number of countries	4			9		
Genome size (Mb)	2.66	2.80	2.97	2.69	2.80	3.12
GC (%)	32.6	32.82	33	32.2	32.73	33
Completeness (%)	98.01	99.44	99.51	98.58	99.45	99.65
Contamination (%)	0.08	0.27	4.05	0.08	0.23	4.15
Number of scaffolds	1	37	416	1	45	248
Scaffolds N50 (bp)	12,677	763,555	2,855,872	121,200	756,086	2,965,341
Number of genes	2,432	2,588	2,856	2,465	2,612	3,021

^*a*^ Global data set.

^*b*^ Local data set.

### Reduced gene content diversity in the global *S. aureus* strains from AD patients

**Fig 1:**
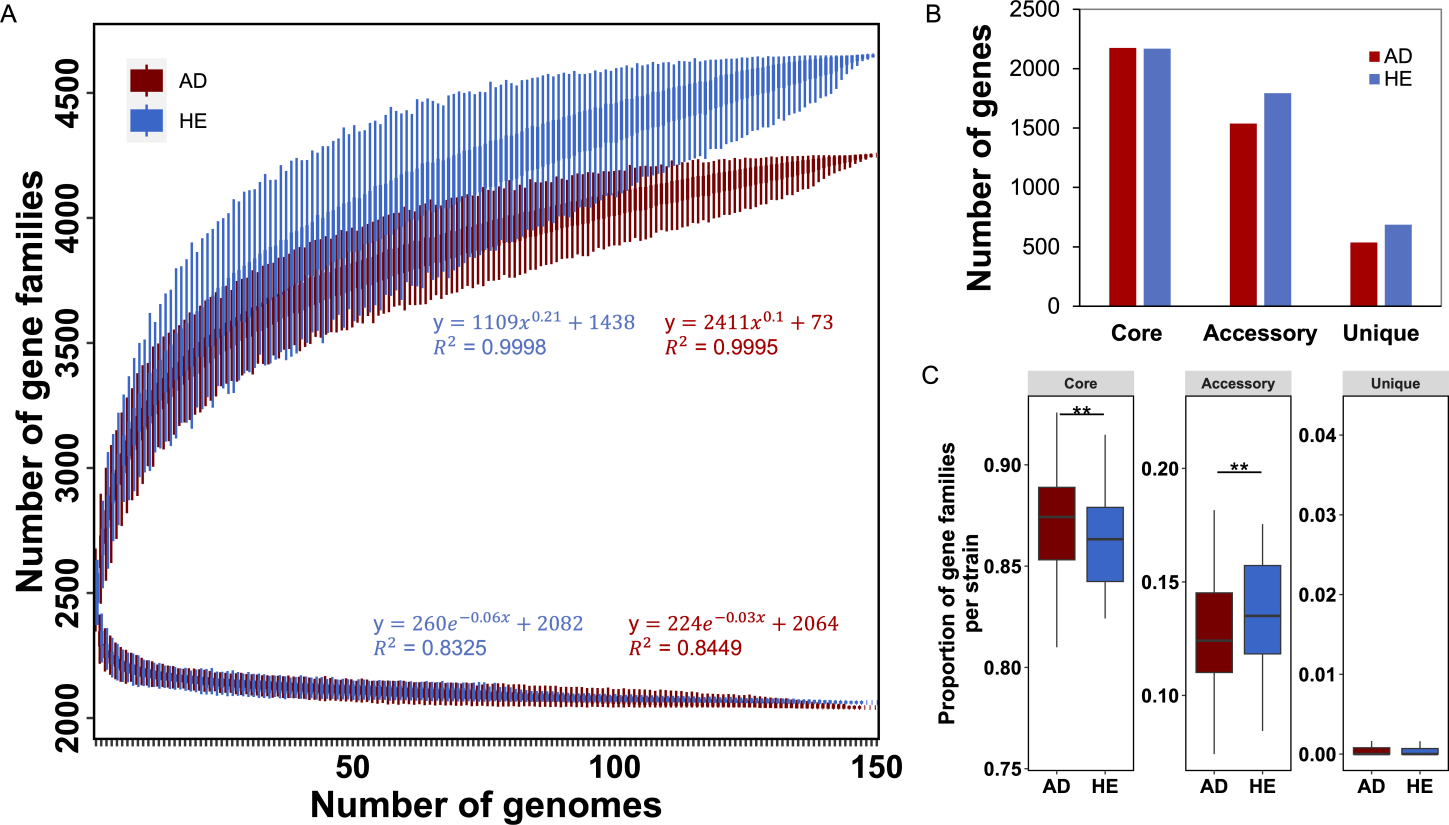
Gene content of the global 300 *S*. *aureus* strains. (**A**) Pan-genome and core genome accumulation curves of strains from AD and HE, separately, as a function of the number of isolates, calculated by PanGP. (**B**) Number of core, accessory, and unique genes for strains from AD and HE, respectively. (**C**) The proportion of gene families within the three gene categories at the strain level (core: in >95% of strains, accessory: in >1 strain but not core, and unique: only in one strain). Statistical significance between AD and HE groups was calculated by the Mann-Whitney U-test. ** *P*-value < 0.01.

To compare the genome differences between the global strains from AD and HE individuals, the gene content diversity of the 300 *S*. *aureus* strains identified an “open” pan-genome and a stable core genome as indicated by the fitting functions, consisting of 41% of core, 40% of accessory, and 19% of unique genes (Fig. S1). When analyzing the pan-genome and core genome of strains from AD and HE separately, the accumulation curves illustrated the pan-genomes for both AD and HE strains increased rapidly and maintained an “open” status, respectively (Fig. 1A). Intriguingly, the increasing rate for strains from HE outpaced strains from AD, achieving a greater pan-genome. However, the core genome size for both groups showed a swift decline, yet most likely stabilizing at a similar size.

To pinpoint genome composition differences between AD and HE, we categorized *S. aureus* genes into core (present in >95% of strains), cloud (present in multiple strains but less prevalent than core), and unique (exclusive to one strain). As shown in Fig. 1B, the core genomes of both groups were notably similar, while strains from HE had more accessory and unique genes than those from AD, accounting for the larger pan-genome size in HE strains. Examining the gene distribution at the strain level revealed a significantly elevated proportion of core genes in AD-associated strains (Mann-Whitney U-test, *P*-value < 0.01, Fig. 1C), hinting at AD-specific selective pressure or homogeneous AD microenvironment fostering strain homogeneity. By contrast, strains from HE were characterized by a significantly higher proportion of accessory genes (Mann-Whitney U-test, *P*-value < 0.01, Fig. 1C). Both groups had a minimal proportion of unique genes.

We also included the commensal skin bacterium *Staphylococcus epidermidis* to compare its genomic diversity with *S. aureus* strains from AD. Typically, *S. epidermidis* strains had a genome size of approximately 2.5 Mb, compared to the 2.8 Mb for *S. aureus* strains (Table S1). *S. epidermidis* exhibited a smaller core genome (~1,830 genes) but a much larger pan-genome (~6,660 genes, Fig. S2A) compared to *S. aureus* strains from AD (~4,260 genes) and HE (~4,650 genes). Further examination revealed a higher number of accessory and unique genes in *S. epidermidis* strains (Fig. S2B). At the strain level (Fig. S2C), *S. epidermidis* strains possessed a significantly lower proportion of core genes and an increased proportion of accessory genes (not statistically significant) and unique genes (statistically significant). These findings suggest that the non-core genes among *S. epidermidis* strains are highly diverse, contributing to the overall greater gene content diversity.

### Greater functional variability in the global *S. aureus* strains from AD patients

**Fig 2:**
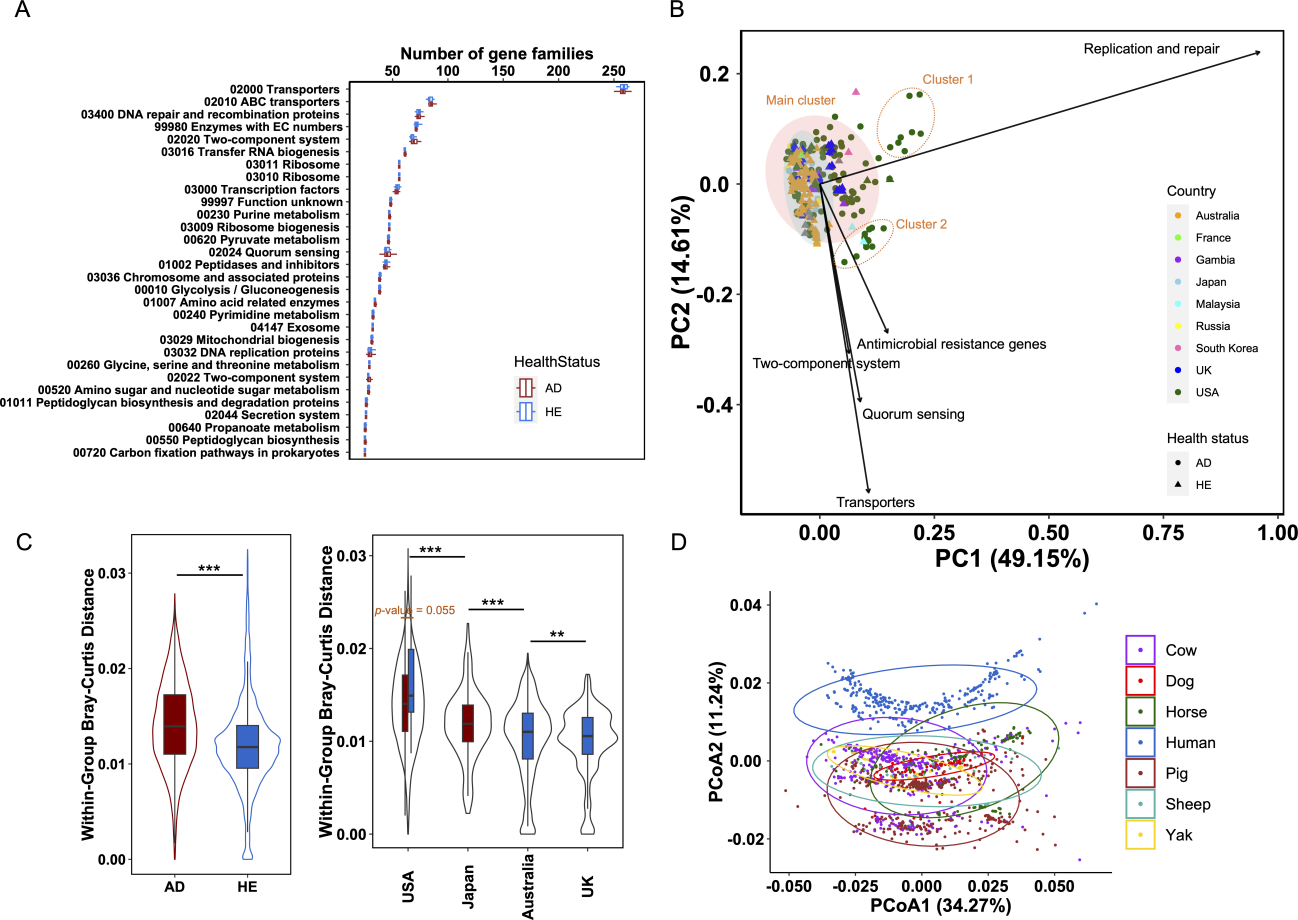
Functional divergence of the 300 global *S. aureus* strains from AD and HE. (**A**) Strain-level abundance of gene families associated with each KEGG functional category. Only the top 30 abundant functions are shown, ranked by median in descending order. (**B**) PCA of the differentiating functions within the global *S. aureus* strains. Only the top five differentiating functions are shown. Colors represent the country of origin and shapes indicate health status. Shaded ellipses represent the 95% confidence interval for the AD (red) and HE (blue) groups. Two additional clusters, Cluster 1 and Cluster 2 are depicted with unfilled dotted ellipses. Variances explained in the two dimensions are expressed in the parenthesis. (**C**) Within-group Bray-Curtis distance in terms of health status and countries of origin. Only the four countries with more than 10 strains are shown. Statistical significance was calculated by the Mann-Whitney U-test for health status and the Kolmogorov-Smirnov test for countries. *P-value < 0.05; **P-value < 0.01; ***P-value < 0.001. (**D**) PCA of *S. aureus* strains isolated from both human and other mammalian hosts, including cow, dog, horse, pig, sheep, and yak, based on the gene abundance table of KEGG functional categories. Colors represent hosts of origin. Ellipses represent the 95% confidence interval for different hosts. Variances explained in the two dimensions are expressed in the parenthesis.

We then profiled the functional repertoire of these strains to investigate whether the trend of reduced gene content in AD strains retained the functional variability. The top five prevalent functions encompassed transporters, ABC transporters, DNA repair and recombination proteins, enzymes with EC numbers, and two-component system, with transporters substantially dominating (Fig. 2A). Principal component analysis (PCA) identified the top five differentiating functions in the strains as replication and repair, transporters, quorum sensing, two-component system, and antimicrobial resistance genes (AMR) on the global level (Fig. 2B). However, AD strains exhibited a significantly broader functional variability than HE strains (Mann-Whitney U-test, *P*-value < 0.001, Fig. 2C). When compared with *S. epidermidis* strains, the top five abundant functional pathways were consistent with those of *S. aureus* strains from AD and HE (Fig. S4A). PCA revealed that *S. aureus* strains from AD showed distinct functional profiles, forming two separate clusters (Fig. S4B). While the top five differentiating functional pathways shared four functions (transporters, replication and repair, two-component system, and quorum sensing), the phosphotransferase system was unique to the comparison between *S. aureus* strains from AD and *S. epidermidis*.

**Fig 3:**
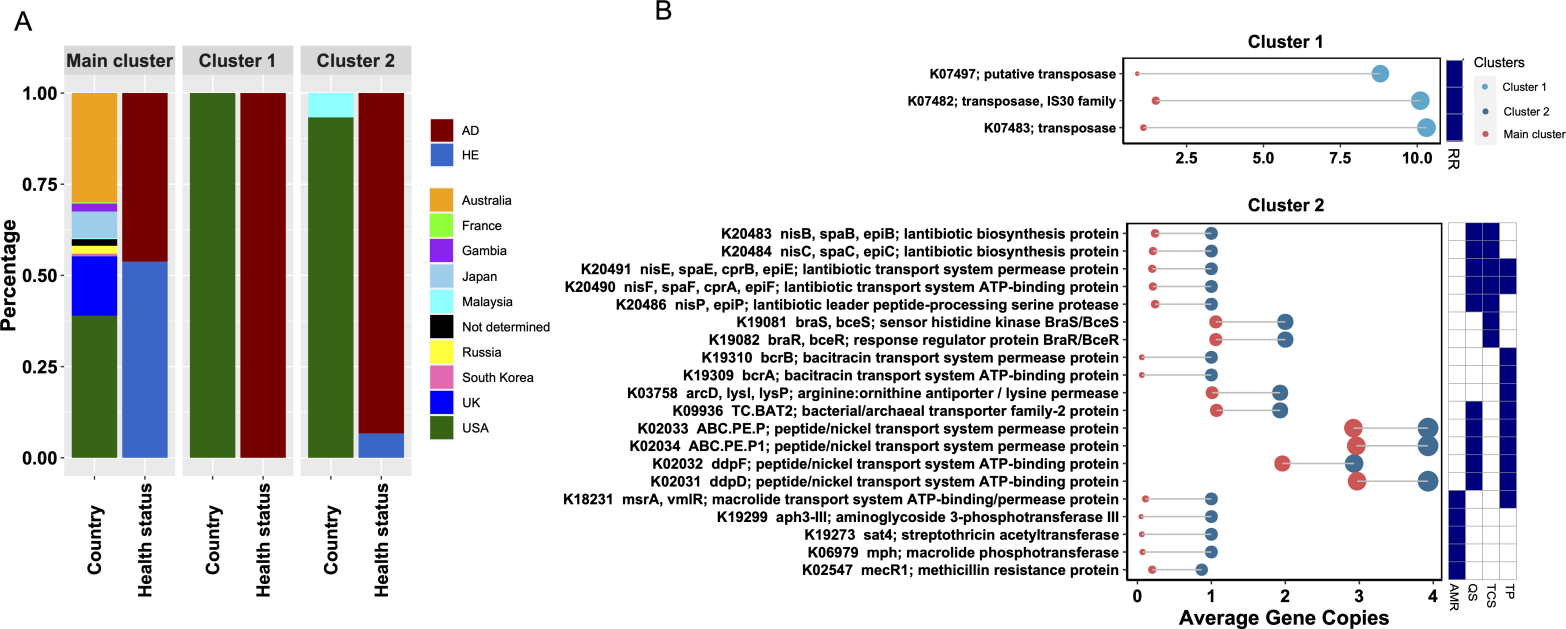
Metadata and functional orthologs diversifying *S. aureus* strains in Cluster 1 and Cluster 2. (**A**) Distribution of countries of origin and health status for the strains of the main cluster, Cluster 1, and Cluster 2. (**B**) Functional genes diversifying the *S. aureus* strains of Cluster 1 and Cluster 2. Dot size indicates the number of average gene copies of strains in the respective cluster, as represented by the X-axis. Colors represent the main cluster (red), Cluster 1 (sky blue), and Cluster 2 (navy blue). The heatmap on the right shows pathways these genes are involved in, with dark blue representing presence. RR, replication and repair; AMR, antimicrobial resistance genes; QS, quorum sensing; TCS, two-component system; TP, transporters.

Interestingly, as depicted in Fig. 2B, the variation in the AD group primarily was mainly linked to strains from the US. Except for the main cluster housing the majority of the strains, two additional distinct clusters were identified that mainly harbored AD strains: Cluster 1 predominantly featured replication and repair, with 9 AD strains; Cluster 2, comprising 13 AD strains and 1 HE strain, was characterized by AMR, transporters, quorum sensing, and two-component system. These two clusters were only slightly affected when strains from AD and HE were clustered separately (Fig. S3). Both clusters were dominant with strains from the US, with only one Malaysian HE strain (Fig. 3A), highlighting the high strain diversity in the US. Significant differences were revealed among strains from different countries including the USA, Japan, Australia, and the UK (Mann-Whitney U-test, all *P*-values < 0.01, Fig. 2C), with the American strains exhibiting the highest diversity. An obvious distinction between AD and HE strains within the US was observed, despite it was not significant (Mann-Whitney U-test, *P*-value = 0.055, Fig. 2C), possibly due to the imbalance in strain numbers (124 AD vs. 6 HE strains).

To further assess the functional association of human-derived *S. aureus* strains and those originating from animals and the potential of zoonosis through host-switching transmission, we performed a PCA on *S. aureus* strains from human and six other mammals (cow, dog, horse, pig, sheep, and yak). The result showed a pronounced distinction between human isolates and those from all other animals (Fig. 2D), implying a distinct origin and evolutionary trajectory of the human-associated strains and limited potential for zoonosis.

### Differential enrichment and acquisition of functional genes drive the diversification of AD-dominant Cluster 1 and Cluster 2

To elucidate what functions drive the differentiation of Cluster 1 and Cluster 2, we examined functional orthologs in the five featured categories (details in Table S2). Compared to the main cluster, Cluster 1 strains demonstrated a 6 to 9-fold higher abundance in three transposases (K07497, K07482, and K07483) representing the KEGG category Replication and Repair (Fig. 3B). Cluster 2 strains were defined by 20 functional orthologs, predominantly found in this cluster or displaying more gene copies than the main cluster. These included five lantibiotic genes (*nisE, nisF, nisC, nisB,* and *nisP*), two *braSR* two-component system genes (*braS* and *braR*), two bacitracin transport genes (*bcrB* and *bcrA*), one arginine antiporter (*arcD*), four peptide/nickel transport genes (ABC.PE.P, ABC.PE.P1, *ddpD*, and *ddpF*), one bacterial/archaeal transporter family 2 protein (TC.BAT2), and five AMR genes (*msrA*, *mph*, *aph3*-III, *sat4*, and *mecR1*). Strikingly, 60% of these genes are related to antibiotic resistance, with the lantibiotic genes, bacitracin transport genes, and AMR genes denoting resistance to lantibiotics, bacitracin, macrolide, aminoglycoside, streptothricin, and methicillin antibiotics, respectively. The *braSR* two-component system and bacterial/archaeal transporter family 2 protein not only act as tools for transporting and regulating a diverse range of molecules but are also involved in processes like stress response and antibiotic resistance. The arginine antiporter and peptide/nickel transport genes are involved in the transport of arginine, an amino acid essential for bacterial growth and survival, as well as peptides and nickel ions, playing a role in nutrient uptake and metal ion homeostasis.

### HGT of the lantibiotic operon in Cluster 2 strains

**Fig 4:**
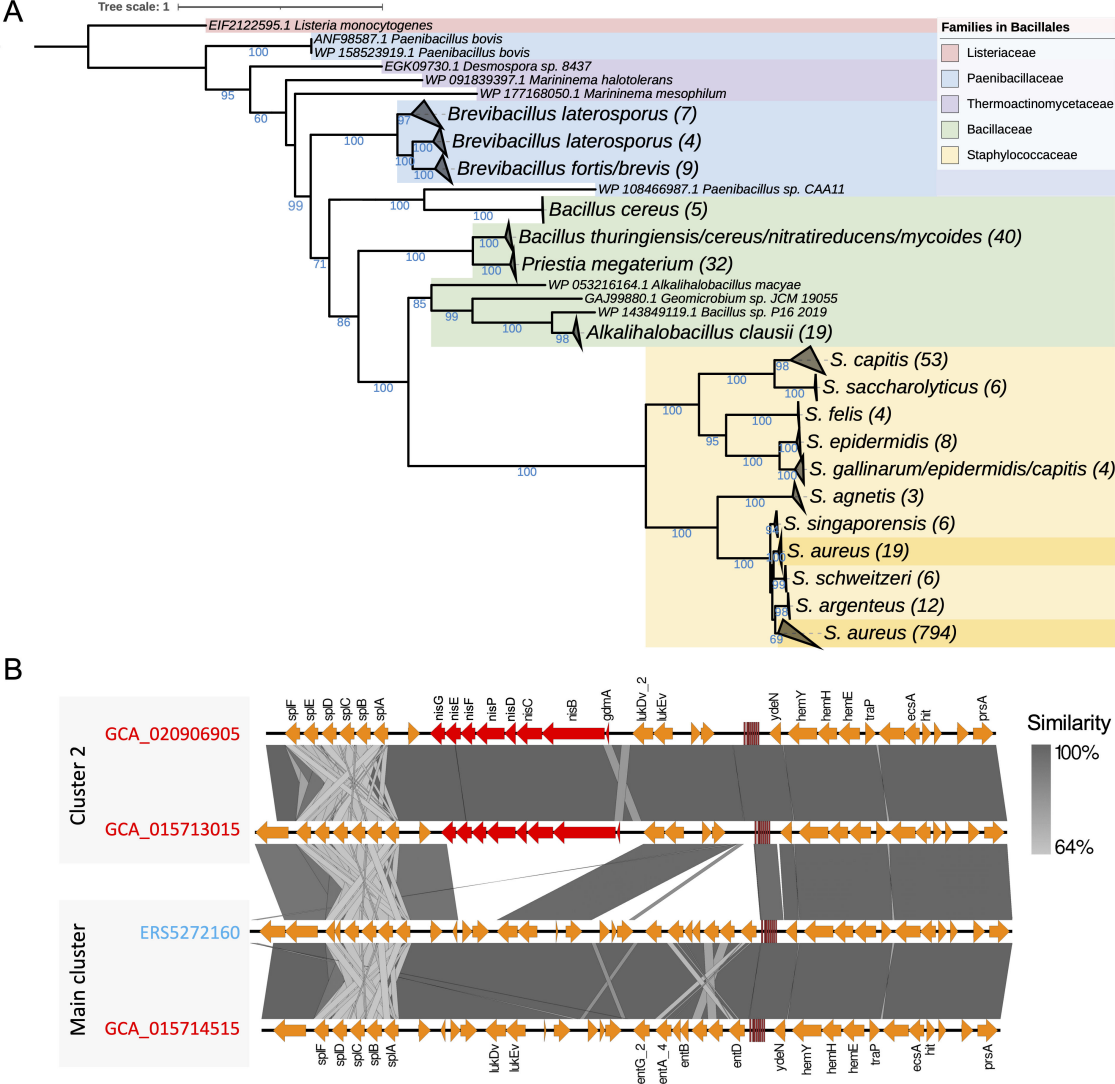
HGT analysis of the lantibiotic operon of human-derived *S. aureus* strains. (**A**) Phylogenetic tree based on the nisB gene of the lantibiotic operon. The colors of the clades show the respective affiliated families under the order Bacillales. *S. aureus* is especially marked in darker yellow. Numbers in the parentheses beside the labels indicate the number of gene sequences included. Numbers beside nodes indicate bootstrap values. (**B**) Synteny of the lantibiotic operon in representative *S. aureus* strains in the main cluster and Cluster 2. The lantibiotic operon is highlighted in red. Gene functions obtained from Prokka are labeled above and below. On the right, AD strains are denoted in red and HE strains in blue. The gradient gray bar at the right denotes the pairwise similarity of the alignment.

To further unravel the mechanism propelling the diversification of Cluster 2, we centered our analysis on the suite of lantibiotic-related genes. Our result already showed that these genes were universally present in Cluster 2 strains with an identical number of gene copies but nearly absent in the main cluster (Fig. 3B). The phylogenetic tree based on the nisin biosynthesis protein *nisB* revealed robust evidence of HGT events of *nisB* across multiple families within the Bacillales order (Fig. 4A). Specifically, the tree topology showed a closer relationship between Bacillaceae and Staphylococcaceae. Within the Bacillaceae clade, multiple genera harbored this gene. However, within the Staphylococcaceae clade, the *nisB* gene was exclusively detected in the single *Staphylococcus* genus. Given the little probability of a single gene spreading from one single genus to multiple disparate families through HGT, it is plausible that certain *S. aureus* strains acquired the lantibiotic operon from other *Staphylococcus* species, which had likely obtained it from members in the Bacillaceae family. Interestingly, the closest Bacillaceae hits included *Alkalihalobacillus clausii*, *Priestia megaterium*, and *Bacillus cereus*, primarily environmental bacteria and widely distributed in nature, implying potential interactions between human-associated and environmental bacteria.

Subsequently, we compared the lantibiotic operon in the genomic sequences of representative *S. aureus* strains from both Cluster 2 and the main cluster (Fig. 4B). The operon, arranged in the *nisA*, *B*, *C*, *D*, *P*, *F*, *E*, and *G* order, was inserted within the genome sequences of Cluster 2 strains, but absent in the corresponding region of strains from the main cluster. This further supports that HGT events led to the acquisition of the lantibiotic operon and propelled the diversification of Cluster 2 strains.

### Local strains mirror global gene diversity but show distinct functional variations between AD and HE groups

**Fig 5:**
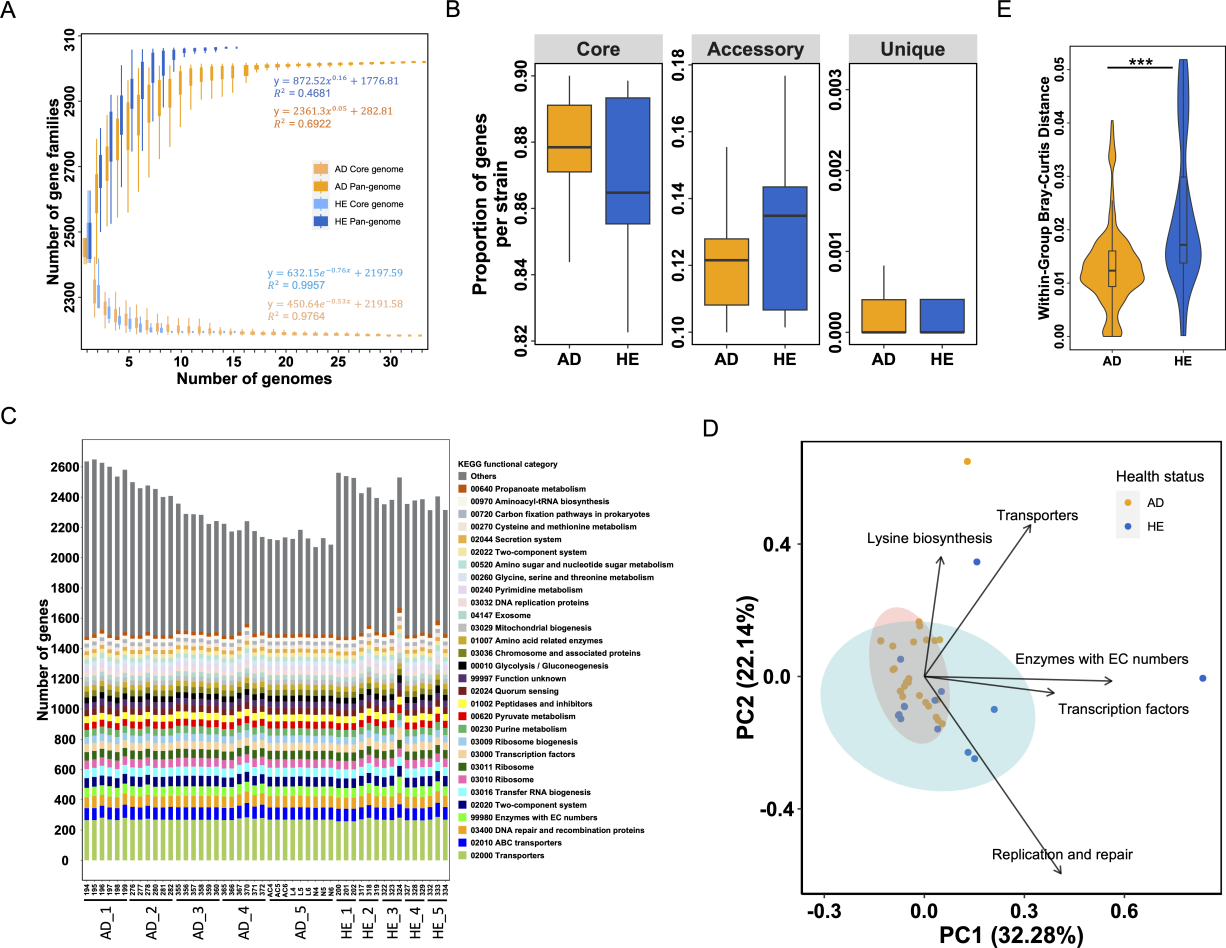
Gene content diversity and functional variation of the 48 local *S. aureus* strains from Germany. (**A**) Core genome and pan-genome accumulation curves of the AD and HE strains, respectively, as a function of the number of isolates. The fitting functions of the curves are shown beside the curves. (**B**) The proportion of gene families at the strain level within the three gene categories (core: in >95% of strains, accessory: in >1 strain but not core, and unique: only in one strain) for both AD and HE strains, respectively. (**C**) Gene abundance of KEGG functional categories for each strain. Only the top 30 abundant functional categories are shown, and the remaining functional categories are shown as Others in gray. The functional categories are ranked by abundance from the bottom to the top. (**D**) PCA based on the abundance of KEGG functional categories. The top five differential functional categories are shown. Ellipses are plotted for the AD (pink) and HE (sky blue) clusters, representing the 95% confidence interval within each group. Variances explained in the two dimensions are expressed in the parenthesis. (**E**) The within-group Bray-Curtis distance of health status. Statistical comparisons between AD and HE groups were performed by the Mann-Whitney U-test. ***P-value < 0.001.

To verify whether global gene content diversity and functional variation patterns hold on a local scale, we utilized 33 *S*. *aureus* strains from AD and 15 from HE sampled from individuals living in Augsburg, Germany. The orthologous gene clustering analysis confirmed that even within a single individual or skin site, strains exhibit genomic diversity. For instance, strains from Patient 150 had nine unique gene families, Patient 9030 had 10, and Patient 119 had 216 accessory genes varying among three of their six strains. The pan-genome of all local strains displayed stability (Fig. S5), with a higher proportion of core genes (64%) than the global strains (41%). The pan-genome and core genome of the local AD and HE strains, respectively, stabilized at approximately 10 genomes (Fig. 5A) and mirrored global patterns with HE strains showing a larger pan-genome than AD strains. At the strain level, local AD strains had more core genes, while HE had more accessory genes. Both groups exhibited minimal unique genes, with no significant difference detected between the two groups across all three gene categories (Fig. 5B).

Functional distribution for each strain showed a clear functional divergence between strains from AD and HE individuals (Fig. 5C), with the most abundant functional categories annotated as transporters, ABC transporters, DNA repair and recombination proteins, enzymes with EC numbers, and two-component system, congruent with the global strains. Surprisingly, PCA delineated that AD strains were more functionally homogeneous than HE strains at the local scale (Wilcoxon rank sum test, *P*-value < 0.001, Fig. 5D and E), contrary to the global data set. The most distinct functional categories were replication and repair, transporters, transcription factors, enzymes with EC numbers, and lysine biosynthesis. Examination of the top 10 differentiating orthologs (Table 2, more differential functions in Table S4) exhibited a large deviation from global ones. Therein, replication and repair featured four transposases (K07497, K07483, K07485, and K07498); transporters featured two genes, including K03893 *arsB* arsenical pump membrane protein and K18104 *abcA*/*bmrA* ATP-binding cassette subfamily B; transcription factors featured two transcriptional regulators, including K21903 *cadC*/*smtB* lead/cadmium/zinc/bismuth-responsive transcriptional repressor and K03892 *arsR* arsenate/arsenite/antimonite-responsive transcriptional repressor; enzymes with EC numbers featured two ion transporter genes, including K01533 *copB* P-type Cu2 +transporter and K01534 *zntA* Zn2+/Cd2+-exporting ATPase. Although transposases play the most important role in differentiation in both the global and local data sets, the other functions related to transporters, transcription factors, enzymes with EC numbers, and lysine biosynthesis were observed only in the local data set. Notably, half of the top ten differential orthologs were involved in the transport and regulation of metal ions.

**TABLE 2:** Top 10 differentiating functional orthologs of the 48 Augsburg strains

Functional categories	Functional orthologs
Replication and repair	Four transposases (K07497, K07483, K07485, and K07498)
Transporters	K03893 arsB arsenical pump membrane protein
	K18104 abcA/bmrA ATP-binding cassette subfamily B
Transcription factors	K21903 cadC/smtB lead/cadmium/zinc/bismuth-responsive transcriptional repressors
	K03892 arsR arsenate/arsenite/antimonite-responsive transcriptional repressors
Enzymes with EC numbers	K01533 copB P-type Cu2+ transporter
	K01534 zntA Zn2+/Cd2+-exporting ATPase

Overall, although the strains obtained from our study site mirrored global strains in gene content, they exhibited unique functional differentiation processes.

## DISCUSSION

In the present study, we conducted a comprehensive whole-genome sequencing-based comparison of 348 *S*. *aureus* strains originating from AD and HE individuals, at both local and global scales. The study illuminated a notable discrepancy in the gene content and functions between AD and HE strains, highlighted unique functional differentiation mechanisms between local and global scales, and revealed evidence of gene enrichment and HGT events as key drivers for *S. aureus* diversification. These observations enhance our understanding of the genomic and functional complexity of *S. aureus* strains associated with AD.

### Pan-genome analysis implicates adaptation and functional diversity

In our study, the gene content analysis revealed that AD-associated strains possess a smaller pan-genome and a significantly higher proportion of core genes at both local and global levels, primarily due to fewer accessory genes in S. *aureus* strains from AD than in strains from HE. These traits signify that the microenvironment of AD skin induces a selective pressure favoring *S. aureus* strains with a highly specialized or adapted set of genes, suggesting a trade-off between genomic economy and functional versatility. This observation also holds true for the comparison between *S. aureus* strains from AD and *S. epidermidis* strains. The adaptation is likely a response to the distinct immune and microbial landscape characteristic of AD, where reduced genomic complexity may confer a selective advantage by streamlining metabolic processes and resistance mechanisms. A recent study from a local clinic in Rome, Italy, reported a similar pattern, with lower gene content in *S. aureus* strains from the AD group (42), further supporting that selective pressure leads to optimization, that is, reduction of the gene repertoire.

Genome reduction is a common adaptive response of microorganisms to diverse microenvironments, enabling them to optimize genetic content for specific ecological niches and challenges (43). This streamlined genetic repertoire enhances fitness by prioritizing essential functions, such as nutrient uptake and stress response. This phenomenon has been observed in other bacteria adapting to specialized environments, reflecting a broader evolutionary strategy to optimize survival under specific conditions. A study found that genome reduction and gene content loss correlate with pathogenicity across various bacterial phyla/species, in which an in-depth investigation of *Streptococcus suis*, a newly identified pathogen that can be transmitted from pigs to humans with multiple transitions between disease and carriage forms, revealed that pathogenicity is consistently associated with reduced genome size within genetic clusters of *S. suis* (44). *Helicobacter pylori* strains, known for causing stomach ulcers and contributing to gastric cancer, demonstrate genomic variation in diverse gastric environments. The hpAfrica2 strain, for instance, exhibits a genome reduction due to the loss of additional genetic elements like the *cag* pathogenicity island as an adaptation to specific gastric environments or dietary habits (45). These findings underscore the dynamic interplay between genome reduction and adaptive responses to environmental challenges.

Given that *S. aureus* is ubiquitous in the environment (46), it raises the intriguing question of whether environmental isolates would form a distinct group compared to *S. aureus* isolates from AD or align more closely with animal isolates. In addition, it prompts the question of whether environmental isolates from locations near hospitals, such as wastewater, would more closely resemble human isolates. Although our study did not directly analyze environmental samples, considering the potential implications of environmental factors—such as proximity to hospitals on the genetic makeup of environmental isolates—could enrich our understanding of microbial adaptation and transmission dynamics across different niches. Such insights are crucial for developing more effective strategies for managing *S. aureus* infections, considering the environmental reservoirs that may contribute to the epidemiology of the disease. Future studies incorporating environmental isolates could provide valuable data to test these hypotheses, ultimately leading to a more comprehensive understanding of the evolutionary trajectories of *S. aureus* in response to varied selective pressures in both clinical and environmental contexts.

HE strains from our sampling sites in Augsburg, Germany revealed a greater functional variation than AD strains, in contrast to global patterns where AD strains displayed greater variability than HE strains. For the local strains, one possible explanation could be the role of transmission dynamics. Strains from HE are often associated with asymptomatic carriage and colonization, which can contribute to higher transmission rates within local populations (47, 48). The increased transmission and exchange of genetic material among strains from HE in the local community may contribute to their higher diversity. It is also plausible that this discrepancy is attributed to the relatively small cohort sizes of local strains analyzed in this study (*n* = 48) and the Rome study (*n* = 38) (42).

*S. aureus* secretes virulence factors that disrupt the epidermal barrier, cytotoxic to various cells like keratinocytes, promote biofilm development for colonization and persistence, and trigger skin inflammation upon colonization (49, 50). However, while some differences in the prevalence of specific virulence genes between isolates from AD and HE have been observed, these differences are often not significant overall (42). Our study aligns with previous studies. While we found some differences in the prevalence of specific virulence genes between *S. aureus* isolates from AD and HE, like hyaluronidase, type VI secretion system ATPase, and acriflavine resistance protein A, these genes were present in only a small number of strains and did not represent significant overall differences. Consequently, our findings suggest that virulence-related genes do not substantially differentiate *S. aureus* isolates from AD and HE, indicating that other factors may play a more critical role in the pathogenicity observed in AD conditions, requiring further investigation.

### Multiple drivers shape *S. aureus* strains

Gene content diversification, potentially driven by natural selection at both micro and macro levels, plays a critical role in bacterial adaptation as it reflects the functional capabilities of a given isolate and the constraints imposed by the corresponding environments (51). In addition to the observed *S. aureus* difference between AD and HE groups at the microscale, this study also unveiled strong disparities between different countries at the macroscale (Fig. 2C), indicating influences from multiple factors like health status and geographical location on *S. aureus* diversification.

A recent study not only validated the positive correlation between *S. aureus* relative abundance and AD severity but also uncovered the significant influences of other patient co-factors such as age, sex, and race on this association (11), which underscores the complex interplay of factors impacting the skin microbiome in AD, in particular *S. aureus* prevalence. Yet, how these factors affect *S. aureus* genetics requires further investigation. Our analysis highlighted a higher diversity of the strains from the US compared to strains from Japan, Australia, and the UK. Previous studies also demonstrate that both health status (27, 37, 52) and geographical location can also significantly influence *S. aureus* strains (32, 53). Recent extensive research revealed that *S. aureus* sequence types (ST) differentially predominated in different countries (32). For example, ST1 is abundant in Europe and Singapore but absent in the US where ST5 is the most prevalent, underscoring the influence of geographical locations on *S. aureus* strains. However, the observed higher diversity in the strains from the US may be attributable to the fact that our data set contains the largest number of strains from the US. We also examined the role of health status within the country, which revealed greater diversity in the HE group for the US cohorts (Mann-Whitney U-test, *P*-value = 0.055, Fig. 2C) and a significantly higher diversity in the HE group for the German local cohorts (Mann-Whitney U-test, *P*-value < 0.001, Fig. 5E). Together with the Rome study that showed a higher gene content in strains of the HE group (42), these findings suggest that both health status and geographical location pronouncedly shape *S. aureus* strain variations.

### Scale-dependent mechanisms and HGT in *S. aureus* diversification

Functional analyses in this study highlight several important aspects of *S. aureus* functional differentiation at both local and global scales. First, “replication and repair” was identified as the most differentiating functional pathway for both the local and global data sets, with transposase enrichment playing a key role. This pathway encompasses various enzymatic activities and molecular mechanisms that collectively contribute to DNA replication, DNA repair, and DNA recombination (54, 55). Interestingly, it has been suggested that various classes of antibiotics widely used in the treatment of staphylococcal infections (56, 57) might induce bacterial dying off through DNA damage (58–60). Transposases may play a role in repairing DNA damage and introducing genetic variation by mediating the excision and repair of damaged DNA regions (61). The enrichment of transposases in AD-dominant Cluster 1 strains indicates their potential involvement in DNA repair and recombination processes to combat the effect of antibiotics used in specific AD patients. Second, while “transporters” emerged as a key differentiating pathway in both local and global data sets, distinct functional orthologs were implicated in each case. In the local strains, relevant genes such as *arsB* (arsenical pump membrane protein) and *abcA* (ATP-binding cassette, subfamily B) genes were prominent, while the global strains featured genes associated with bacitracin (*bcrA* and *bcrB*), arginine (*arcD*), and peptide/nickel transport (ABC.PE.P, ABC.PE.P1, *ddpD*, and *ddpF*). These genes can not only power the translocation and regulation of metal ions but also a variety of structurally diverse antimicrobial compounds from their cells, contributing to bacterial survival and resistance against antibiotics (62, 63). Third, a notable proportion of metal-related genes, including *cadC*, *arsR*, *copB*, and *zntA* were among the top differentiating orthologs in the local data set, which underlines the significant role of metal transport and regulation for the differentiation of the local strain. By contrast, the global data set revealed five AMR genes against macrolide, aminoglycoside, streptothricin, and methicillin, alongside the lantibiotic operon as the important orthologs driving the diversification of the AD-dominant Cluster two strains from the US, underscoring the importance of AMRs in differentiating *S. aureus* strains at the global scale and the high antibiotic prescribing rate in the US (64). However, it remains unclear why genes coding metal ions uptake are so significant in differentiating the local strains. Further studies are needed to elucidate the specific functions and mechanisms. Overall, these results highlight geographical scale-dependent mechanisms of *S. aureus* divergence.

The lantibiotic operon is responsible for the production of lantibiotics, also known as the nisin operon, and typically contains a cluster of genes involved in the biosynthesis and regulation of the lantibiotic nisin (65). Evidence of HGT of the lantibiotic operon from Bacillaceae, which are widely distributed in nature (66), suggests HGT as a common mechanism for the acquisition of AMR genes and highlights the potential interactions between human-associated and environmental bacteria in shaping microbial diversity and adaptation. The mechanisms of evolution and diversification of lantibiotic biosynthesis pathways thus contribute to our understanding of *S. aureus* adaptation and the spread of antimicrobial resistance. Phage transduction has been suggested to be the dominant HGT mechanism in *S. aureus* (67). However, to what extent phages contribute to *S. aureus* gene repertoire and thus differentiation needs further investigation.

### Conclusions

Our research provides a profound understanding of the genomic variations in *S. aureus* strains linked to AD. Both local and global strains reveal a trend toward adaptive evolution in the specific AD microenvironment, with AD-associated strains possessing a smaller pan-genome and a greater share of core genes. Key functional categories like replication and repair, as well as transporters, play a central role in diversifying these strains. The increased presence of transposases suggests them as important drivers for *S. aureus* diversification at both global and local scales. The enrichment of metal uptake-related genes in local strains compared to antimicrobial resistance genes in global strains highlights the nuanced roles of metal transport and antibiotic resistance in the scale-dependent diversification processes. The detection of HGT events further underscores the genetic interaction between human-associated and environmental bacteria. Our findings offer critical implications for clinical perspectives. The recognition of key functional categories and the presence of transposases in these strains underscores potential targets for novel antimicrobial interventions. Future studies should focus on the functional analysis of these genetic variations to elucidate their specific roles in AD. Longitudinal studies involving a geographically diverse range of AD patients could provide deeper insights into the dynamic interaction between *S. aureus* strains and the host. In addition, exploring the environmental factors influencing the genetic diversification of these strains could further clarify their adaptability and survival mechanisms in different microenvironments.

## MATERIALS AND METHODS

### Origin of *S. aureus* genomes

The qualities of all genomes used in this study were assessed using CheckM v1.1.3 (68). Detailed information on all human-derived strains is included in Table S1. Default parameters were used for all tools described below unless otherwise specified.

A total of 300 genomes of *S. aureus* strains from AD and HE were collected from public databases representing a broad global spectrum of sites where strains were originally isolated. For *S. aureus* strains from AD, we searched for *S. aureus* genomes from the NCBI BioSample database using the keywords “Atopic dermatitis and *Staphylococcus aureus*” (1,707 results, March 3, 2023); subsequently, these results were exported in the format “Accessions list”; these accession numbers were matched with the metadata of all *S. aureus* assemblies downloaded from the NCBI Genome database (https://www.ncbi.nlm.nih.gov/genome/browse/#!/prokaryotes/154/). Furthermore, the matched assembly accession numbers were used to retrieve *S. aureus* genome sequences using ncbi-genome-download v0.3.1 with parameters “bacteria --section genbank --formats fasta --flat-output” (69) (156 assemblies downloaded). After quality control (completeness >98%, contamination <3%), 150 *S*. *aureus* genomes from AD were retained for further analysis.

For *S. aureus* strains from HE, since there were not enough *S. aureus* assemblies with qualified metadata available on the NCBI Genome database, we searched the BioSample database with the keywords “*Staphylococcus aureus* and healthy skin and *Homo sapiens*” (364 results, March 3, 2023). The search results were exported and the Sequence Read Archive (SRA) accession numbers were used to download whole-genome sequencing raw read samples using Prefetch v2.8.0 (70), obtaining 338 SRA samples. These samples were then processed to split forward and reverse reads using Fasterq-dump v2.8.0 (70) and manually assembled using Spades v3.13.0 with parameters “--careful -k 55,77,99,127 --cov-cutoff auto” (71). After quality control, 209 assemblies were retained. To maintain a comparable number of genomes to the AD strains for further analysis, we subsampled 150 *S*. *aureus* genomes from HE.

In addition, a set of 48 *S*. *aureus* strains were isolated from five AD patients and five healthy individuals within a study cohort established at the Klinikum Augsburg in Germany. For *S. aureus* strains from AD, we isolated *S. aureus* strains from both the nose and skin (three strains for each) of five AD patients. Specifically, patient ID 291 provided three strains from non-lesional antecubital fossa skin, lesional hand skin, and nose, respectively. This resulted in 33 strains from AD. For strains from HE, we isolated three strains only from the nose of each of the five healthy controls due to the very low abundance of *S. aureus* on healthy skin, resulting in 15 strains from HE. To minimize duplications among the 48 strains, we performed whole-genome sequencing using long-read technology, yielding high-quality assemblies with 44 complete chromosomes and enabling precise strain-level differentiation. In addition, we constructed a phylogenomic tree (not shown here) and analyzed orthologous gene clustering, which identified phylogenetic distance and unique gene families among strains from the same person or skin site. These results proved the strains within person or within skin site are not totally identical. The detailed protocol including isolation, culturing, identification, sequencing, and assembly (including accession numbers) of these *S. aureus* strains has been previously described in reference (41, 41). The sampling was approved by the ethics committee of the Technical University of Munich (112/16 S and 187/17 S).

To investigate the association of human-derived and animal *S. aureus* strains, *S. aureus* assemblies from other mammalian animals were collected from the BV-BRC database (https://www.bv-brc.org, August 15, 2023). We selected *S. aureus* genomes isolated from cows, dogs, horses, pigs, sheep, and yaks due to their high availability which could ensure robust results. The accession numbers of the selected strains were used to retrieve genome sequences using Batch Entrez (https://www.ncbi.nlm.nih.gov/sites/batchentrez). Genomes with abnormal sizes deviating from the normal size of *S. aureus* at ~2.8 Mb or with high contamination (>10%) were excluded, ensuring a comprehensive selection of high-quality *S. aureus* genomes for our study. After quality control, we included 333 strains from cows, 56 strains from dogs, 145 strains from horses, 391 strains from pigs, 34 strains from sheep, and 83 strains from yaks for the analysis. Detailed information on all mammalian strains is included in Table S3.

### Collection of *Staphylococcus epidermidis* strains

To compare *S. aureus* isolates obtained from AD with other commensal skin bacteria like *S. epidermitis*, we collected the genomes of *S. epidermidis* strains from the BV-BRC database (https://www.bv-brc.org, 1,495 genomes available, 28 May, 2024); then we selected the genomes derived from human skin as the isolation source (341 qualified), of which the GenBank accessions were used to download whole-genome sequences using Batch Entrez (https://www.ncbi.nlm.nih.gov/sites/batchentrez). To maintain a comparable number of genomes to the *S. aureus* strains from AD for further analysis, we subsampled 150 *S*. *epidermidis* genomes with completeness >99% and contamination <1%. For the subsequent genomic and functional analyses, the same methods were used as described for *S. aureus* strains from AD and HE strains. Detailed information on all *S. epidermidis* strains is included in Table S1.

### Pan-genome and core genome calculation

Open reading frames (ORFs) were predicted using Prodigal v2.6.3 (72). Genes of all *S. aureus* and *S. epidermidis* genomes were compared with each other using BLASTP version 2.12.0 + with commands -evalue 1e-5 (73). BLAST results were then filtered to a percent identity of 70% and query coverage of 75% (74). Finally, orthologous gene clustering was performed using MCL version 14-137 with an inflation value of 2 (75). Based on the gene presence-absence table obtained from MCL, pan-genome and core genome curves and fitting functions were calculated using PanGP v1.0.1 with Distance Guide as the sample algorithm (76). Core genes were defined as those that are present in more than 95% of strains; cloud genes as those that are present in more than one strain but not core; unique genes were those that are only present in a single strain.

### Functional annotation

Gene functions of *S. aureus* and *S. epidermidis* strains were annotated using GhostKOALA version 3.0 (77) searching the “genus_prokaryotes + viruses” database online (August 15, 2022), complemented the annotation using Prokka v1.14.6 (78) with settings “--genus Staphylococcus --gram + --evalue 1e-05.” The gene abundance for each KEGG functional category in each genome was calculated using a custom Python script with genes functionally unannotated assigned into “unassigned,” based on which the PCA analysis of differentiating functions for all *S. aureus* data sets was performed with R package Vegan v2.5–6 (79). The additional two clusters were singled out to compare their average gene abundance in selected functional categories with the main cluster.

### Phylogenetic analysis

The *nisB* gene, coding for the lantibiotic biosynthesis protein, was used for the phylogenetic analysis due to its length in the lantibiotic operon, which can provide a higher resolution in analysis and a higher likelihood of detecting HGT events. All *nisB* gene sequences of the *S. aureus* strains from this study were extracted by blasting all genes to the known *nisB* gene (WP_001092605) using BLASTP v2.12.0 + with 70% of identify and 75% of coverage (73). Then all extracted *nisB* genes were blasted against the NCBI Non-redundant database online (September 14, 2022) and the top 1,000 best hits were outputted for further processing. Subsequently, the outputted *nisB* gene sequences were aligned using Mafft v7.310 (80) utilizing the parameters “--maxiterate 1000 –localpair” to maximize iterative refinement. All gap positions in the alignment with gaps in 50% or more of the sequences were removed using TrimAI v1.4.rev22 (81) with –gt 0.5. This curated alignment was used to infer the phylogenetic tree using RAxML version 8.2.11 with the PROTGAMMAAUTO model and 200 bootstraps (82). The phylogenetic tree was edited with iTOL (83).

The lantibiotic operon synteny was visualized in Easyfig 2.2.5 (84) based on the annotation files in gbk format outputted by Prokka v1.14.6 (78).

### Statistical analysis

Statistical analyses were conducted starting with assessing the normality of the data distribution using either the Shapiro-Wilk test or the Kolmogorov-Smirnov test when the observations exceed 5,000. Subsequently, the Welch two-sample *t*-test (normal distribution) or non-parametric Mann-Whitney U-test also called the Wilcoxon rank sum test (non-normal distribution) was employed to evaluate the statistical significance of observed differences. Any *P*-value < 0.05 was considered indicative of statistical significance.

## Data Availability

The genome accession numbers of *S. aureus* strains used in this study have been included in Supplementary Tables.

## SUPPLEMENTAL MATERIAL

Supplemental figures spectrum.00571-24-s0001.pdf Fig. S1 to S5.

Supplemental material spectrum.00571-24-s0002.tiff Graphical abstract.

Table S1 spectrum.00571-24-s0003.xlsx Metadata of *Staphylococcus aureus* and *S. epidermitis* strains from human.

Table S2 spectrum.00571-24-s0004.xlsx Metadata of Cluster 1 and Cluster 2.

Table S3 spectrum.00571-24-s0005.xlsx Metadata of *Staphylococcus aureus* from animals.

Table S4 spectrum.00571-24-s0006.xlsx Top 20 differentiating functions of the Augsburg strains.

ASM does not own the copyrights to Supplemental Material that may be linked to, or accessed through, an article. The authors have granted ASM a non-exclusive, world-wide license to publish the Supplemental Material files. Please contact the corresponding author directly for reuse.
