# Supplementary material for: Genomic and functional divergence of Staphylococcus aureus strains from atopic dermatitis patients and healthy individuals: insights from global and local scales
Source: Microbiol Spectr. 2024 Aug 20;12(10):e00571-24. doi: 10.1128/spectrum.00571-24 (PMC11448032; doi:10.1128/spectrum.00571-24)
Supplement: Supplemental figures — Fig. S1 to S5. [file spectrum.00571-24-s0001.pdf]

Genomic and functional divergence of *Staphylococcus aureus* strains from atopic dermatitis patients and healthy individuals: insights from global and local scales

Zhongjie Wang[a],# Claudia Hülpüsch[b], Bärbel Foesel[a*], Claudia Traidl-Hoffmann[b,c,d], Matthias Reiger[b] & Michael Schloter[a,e]

[a]Research Unit for Comparative Microbiome Analysis, Helmholtz Munich, German Research Center for Environmental Health, Neuherberg, Germany

[b]Institute of Environmental Medicine, Helmholtz Munich, German Research Center for Environmental Health, Neuherberg, Germany

[c]Environmental Medicine, Faculty of Medicine, University of Augsburg, Augsburg, Germany

[d]CK CARE, Christine Kühne Center for Allergy Research and Education, Davos, Switzerland

[e]Chair of Environmental Microbiology, TUM School of Life Sciences Weihenstephan, Technical University of Munich, Freising, Germany

Running title: Diversities of *S. aureus* in AD Patients vs. Healthy

*Present address: Bärbel Foesel, Research Unit Molecular Epidemiology, Institute of Epidemiology, Helmholtz Munich, German Research Center for Environmental Health, Neuherberg, Germany

Author order was determined based on contribution.

Supplementary figures

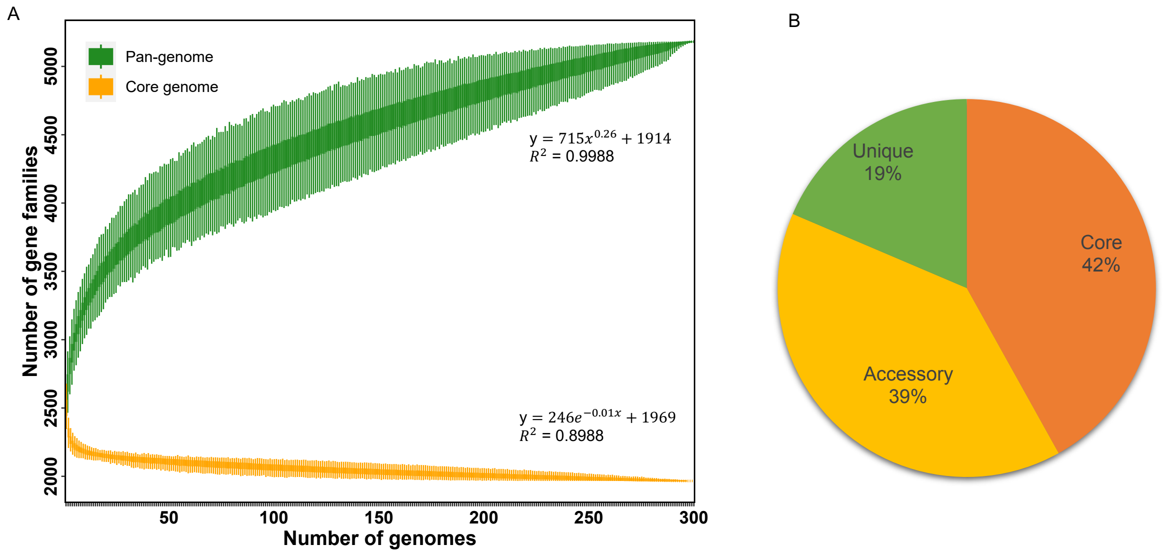

Figure S1. (A) Pan-genome and core genome accumulation curves of the global 300 *S. aureus* strains from AD and HE as a function of the number of isolates, calculated by PanGP. (B) Pie chart of the percentage of gene families within the three gene categories (core: in >95% of strains, accessory: in > 1 strains but not core, and unique: only in 1 strain) for the 300 strains from AD and HE in the global dataset.

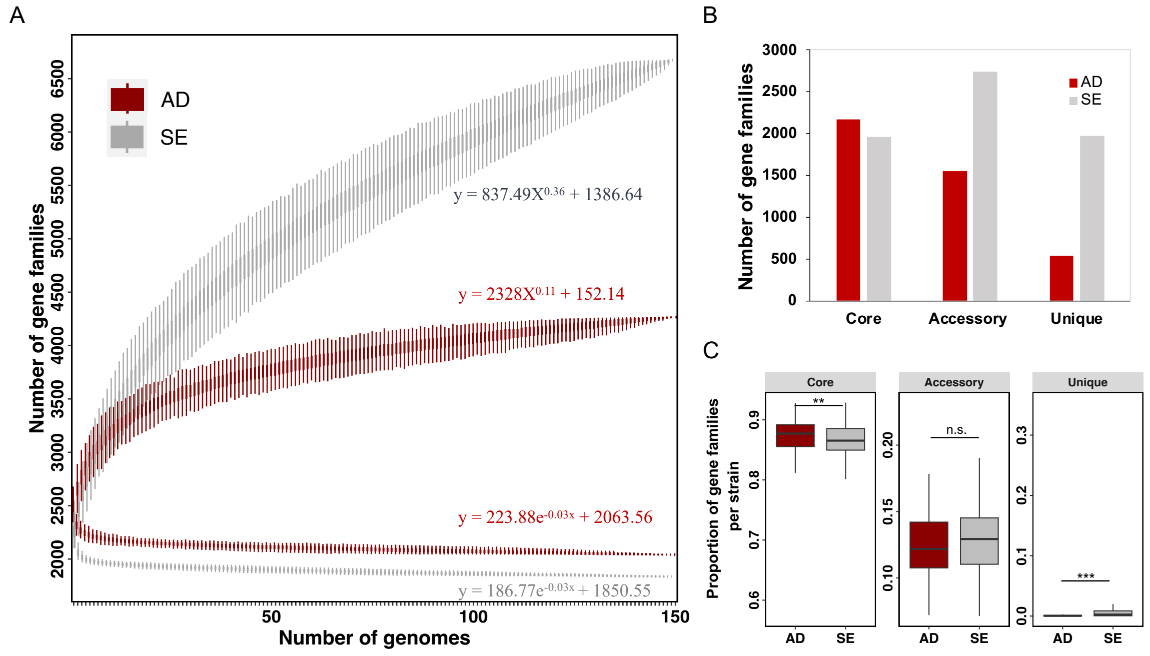

Figure S2. Gene content of the 150 *S. epidermitis* (SE) strains and 150 *S. aureus* strains from AD. (A) Pan-genome and core genome accumulation curves of strains from AD and SE, separately, as a function of the number of isolates, calculated by PanGP. (B) Number of core, accessory, and unique genes for strains from AD and *S. epidermitis*, respectively. (C) Proportion of gene families within the three gene categories at the strain level within each group (core: in >95% of strains, accessory: in > 1 strains but not core, and unique: only in 1 strain). Statistical significance between AD and SE groups was calculated by the Mann-Whitney U-test. * *p*-value < 0.05; ** *p*-value < 0.01; *** *p*-value < 0.001; n.s. = not significant.

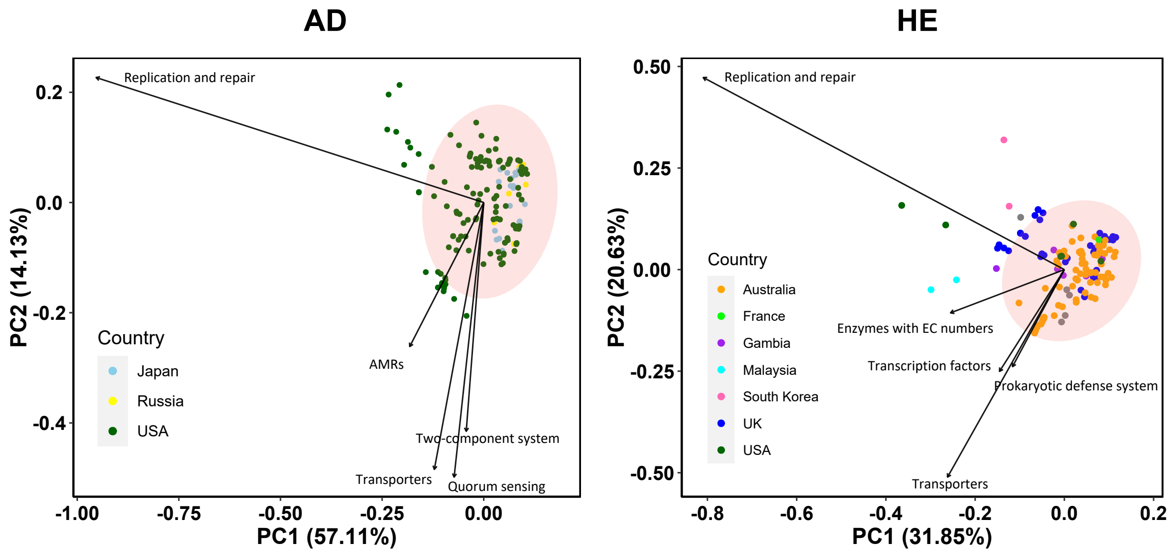

Figure S3. PCA analysis of differentiating functions within the global *S. aureus* strains for AD and HE groups, separately. Only the top 5 differentiating functions are shown. Colors represent the country of origin. Shaded ellipses represent the 95% confidence interval for the AD and HE groups. Variances explained in the two dimensions are expressed in the parenthesis.

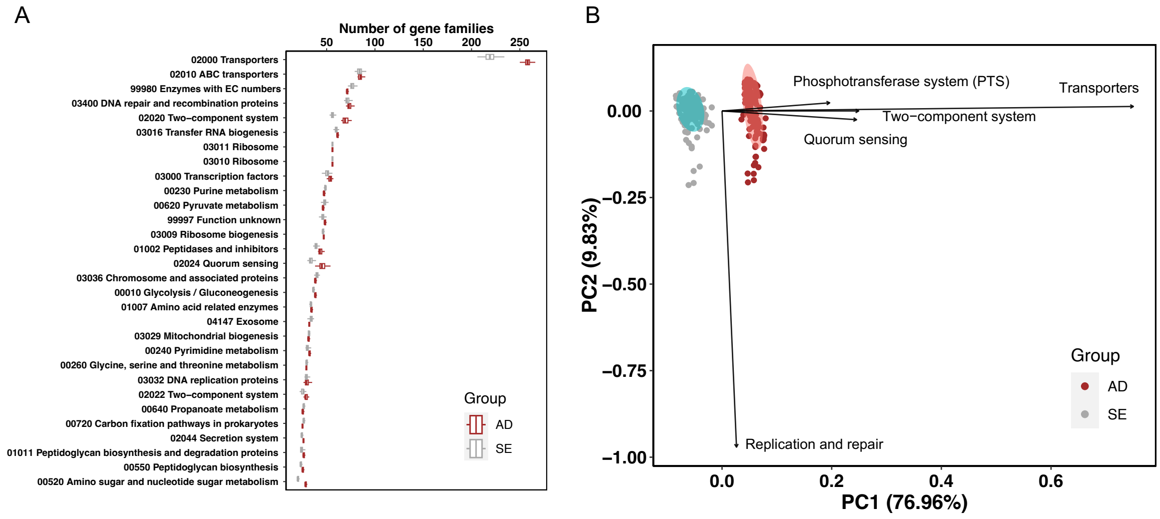

Figure S4. Functional annotation of the 150 *S. epidermitis* (SE) strains and 150 AD strains. (A) Abundance of gene families associated with each KEGG functional category. Only the top 30 abundant functions are shown, ranked by median in descending order. (B) PCA analysis of the differentiating functions between *S. epidermitis* and *S. aureus* strains from AD. Only the top 5 differentiating functions are shown. Colors represent the species. Shaded ellipses represent the 95% confidence interval for the AD (red) and SE (blue) groups. Variances explained in the two dimensions are expressed in the parenthesis in the axis.

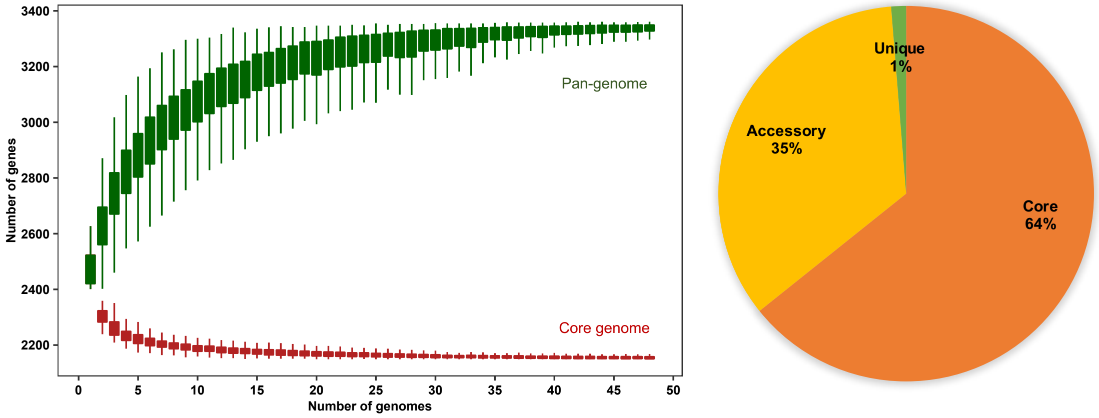

Figure S5. (A) Pan-genome and core genome accumulation curves of the local 48 *S. aureus* strains from AD and HE isolated from Augsburg. The fitting functions of the curves were not shown as both curves remain stable with the increase of strains. (B) Pie chart of the percentage of gene families within the three gene categories (core: in >95% of strains, accessory: in > 1 strains but not core, and unique: only in 1 strain) for strains from AD and HE in the local dataset.
