# Supplementary figures and images for: Genomic and functional divergence of Staphylococcus aureus strains from atopic dermatitis patients and healthy individuals: insights from global and local scales
Source: Microbiol Spectr. 2024 Aug 20;12(10):e00571-24. doi: 10.1128/spectrum.00571-24 (PMC11448032; doi:10.1128/spectrum.00571-24)

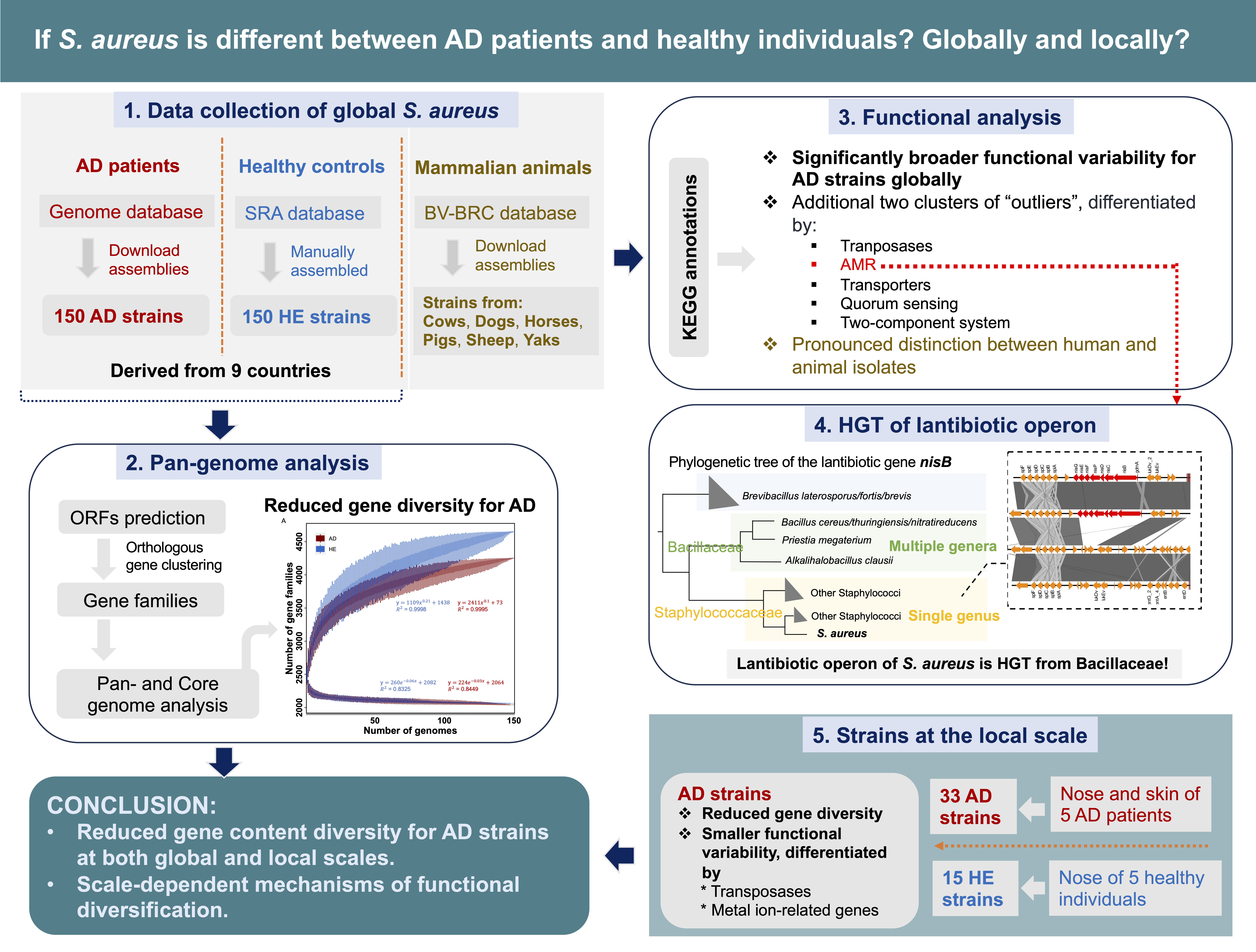

Supplement: Supplemental material — Graphical abstract. [file spectrum.00571-24-s0002.tiff]
